# Factors associated with citations of articles on circular economy in the Web of Science: modeling for main publishers

**DOI:** 10.3389/frai.2023.1217210

**Published:** 2023-09-29

**Authors:** Carlos Alberto Minchón-Medina, Daphne Jannet Timaná-Palacios, Aldo Alvarez-Risco, Shyla Del-Aguila-Arcentales, Jaime A. Yáñez

**Affiliations:** ^1^Departamento de Estadística, Facultad de Ciencias Físicas y Matemática, Universidad Nacional de Trujillo, Trujillo, Perú; ^2^Universidad Tecnológica del Perú, Lima, Perú; ^3^Escuela de Posgrado, Universidad San Ignacio de Loyola, Lima, Perú; ^4^Vicerrectorado de Investigación, Universidad Norbert Wiener, Lima, Perú

**Keywords:** circular economy, modeling, articles, Web of Science, citations

## Abstract

**Introduction:**

The publication of articles on the circular economy has different associated factors to explain the citations registered in the Web of Science.

**Method:**

Articles from the publishers Elsevier, MDPI, Taylor & Francis, Wiley, and Springer Nature were evaluated.

**Results:**

It was expected that the older the article was, the more citations it had received, but this was not always the case. It was also recognized that there was a lower number of citations if the articles were too large or if they had too many references.

**Discussion:**

This analysis helps to establish the factors that must be addressed in order to publish in journals that have a high citation rate. Conclusion: Based on speci?c articles and with speci?c references, it will be possible to increase the probability of citations.

## Introduction

When conducting and publishing research, authors are always looking to achieve citations as it is an indicator of the impact of their research efforts (Castillo et al., 2007; Petersen et al., 2014; Herman, 2018; Massucci and Docampo, 2019). A topic that has been gaining more and more interest is the circular economy, which has been described from different areas and disciplines such as packaging (Castillo-Benancio et al., 2022; Silva and Pålsson, 2022), firm performance (Alvarez-Risco et al., 2021; Santa-Maria et al., 2022; Triguero et al., 2022), plastic management (Alvarez-Risco et al., 2020; Johansen et al., 2022; Rosenboom et al., 2022), foods (Del-Aguila-Arcentales et al., 2022; Durkin et al., 2022; Tamasiga et al., 2022; Zhang et al., 2022), supply chain (Alvarez-Risco et al., 2022; Berlin et al., 2022; Khan and Abonyi, 2022; Lavelli and Beccalli, 2022), water (Kakwani and Kalbar, 2020; Chen et al., 2022; Khoury et al., 2023), recycling (Bongers and Casas, 2022; De-La-Torre-Jave et al., 2022; Islam and Iyer-Raniga, 2022; Neumann et al., 2022), and globalization (Ibn-Mohammed et al., 2021; Anderson-Seminario and Alvarez-Risco, 2022; Aublet-Cuvelier et al., 2022).

There are several factors that can influence the number of citations that articles on the topic of the circular economy receive in the Web of Science database. The main factors recognized include the quality of the research, as articles that are well designed and make a significant contribution to the circular economy field are more likely to be cited. Relevance to the field is recognized because articles that address important issues or provide new knowledge in the circular economy field are more likely to be cited. Also, timeliness is recognized because articles that are published at a time when there is a high level of interest in the topic of the circular economy are more likely to be cited. Journal reputation is recognized because articles that are published in respected, high-impact journals are more likely to be cited. Also decisive is the reputation of the author because articles written by researchers with a strong reputation in the field of the circular economy are more likely to be cited. Additionally, the length and format of the article is an influential aspect, as complete and wellstructured articles are more likely to be cited. Finally, the use of visual and graphical elements is also recognized because articles that use visual aids, such as graphs and tables, to communicate their conclusions are more likely to be cited by future researchers. Despite the reasonableness of these factors, a deeper and more systematic analysis is needed to determine precisely which factors really influence the citation of publications.

## Materials and methods

### Design and population

The quantitative study was conducted using bibliometric data of articles from the Web of Science (WoS) database, using “circular economy” as the search criteria of article-type documents; the term was specified in the title, keywords, abstract or remaining text. It was refined by Citations Topics Meso: 6,115 Sustainability Science articles, published from 2010 to October 2022. The WoS was considered precisely because it united the concepts of sustainability and the circular economy, which seek to impact the environment as little as possible. The downloaded study population comprised 4,153 articles, with no additional search restrictions in terms of type of access, WoS categories, research areas, or other. Some articles did not present the year of publication, and it was necessary to resort to the journals of the publishers in which they were published to complete this information. The simple article search protocol will allow replication of the process and serve as a basis for future research. Emphasis was placed on publishers to compare scientific production and evaluate the factors associated with the impact on the number of citations, where the published articles belong to one and only one publisher. The main publishers own enormous numbers of journals worldwide.

### Procedure

The bibliometric data for this research were retrieved from the WoS database, with the last publication date being 31 October 2022, using the search criteria indicated above. The bibliometric data were exported from the WoS in Excel format, by selecting Record Content on a full record. This system allowed the necessary columns to obtain the following study factors directly from each article or by means of Excel functions:

- Publisher
- Timed cited
- Number of pages
- Number of authors
- Number of research areas
- Number of authors keywords
- Funding orgs (1: Specifies financial institutions, 0: Not specified)

The publishers with the most publications on the circular economy were: Elsevier, MDPI, Wiley, Springer Nature, and Taylor & Francis. The journal in which the article was published or its impact index was not considered as a variable because journals participate in several areas. On the other hand, the authors focused their interest on editorials for the design of policies in the publisher's journals. The analysis, using IBM SPSS Statistics version 27 (https://www.ibm.com/es-es/products/spss-statistics), comprised firstly a comparison of the annual production between publishers in the period 2010–2021, using the classical ANOVA to compare the mean production and Tukey's test, but in addition, negative binomial regression was used for count data. Furthermore, the annual evolution of scientific production was analyzed using logarithmic regression models, plotted for each publisher separately and for all publications downloaded from the WoS. The procedures were carried out in Excel which allowed in the same procedure the estimation of the equation and editing of the figures of the growth of scientific production in the circular economy field. The characteristics of the articles published in the period of 2010 to October 2022 were reported through descriptive statistics: minimum (Min), maximum (Max), mean (Mean), standard deviation (Std. Deviation), and non-citation (Non citation, %), for each publisher. Non-citation corresponds to articles in which the WoS provides a blank field, assuming “zero” citations. No tracking was done because the source of the data would not be the WoS alone.

### Data analysis

Factors associated with article citations and non-citations were analyzed using negative binomial regression models (Didegah, 2014; Ajiferuke and Felix, 2015) using Stata version 16 (StataCorp. Stata Statistical Software: Release 16. College Station, TX: StataCorp LLC; 2019). Due to the presence of overdispersion, Poisson regression models were discarded, evaluated using the chi-square test (Hilbe, 2011). Also, given the high percentage of uncited articles from each publisher, the zero-inflated negative binomial regression model (Didegah, 2014; Ajiferuke and Felix, 2015) was also considered. The models included robust estimates of standard deviations, which is why a column was included in the tables. Finally, the models were compared using the Vuong test and the Akaike (AIC) and Bayesian (BIC) information criteria and estimates (Hilbe). Stata features specific menus for both count responses and generalized linear models (GLMs).

## Results and discussion

### Scientific production

The scientific production on the circular economy totaled 4,153 articles; 3,060 corresponded to the period 2010–2021, and 1,093 to 2022. The annual production published in journals of the publishers in the period 2010–2021 is shown in Table 1; the top five publishers were Elsevier (1,326 articles), MDPI (640 articles), Wiley (179 articles), Springer Nature (171 articles), and Taylor & Francis (126 articles). In mean terms, the publishers presented annual differences according to ANOVA (*p* = 0.20 < 0.05), mainly due to the higher number of publications in Elsevier (110.5 ± 155.4 articles/year) according to Tukey's test. Other publishers were not included in this analysis.

**Table 1:** Scientific production on the circular economy published on the Web of Science according to main publishers.

	**Publisher**						
**Year**	**Elsevier**	**MDPI**	**Wiley**	**Springer Nature & Taylor**	**Francis**	**Other**	**WoS**
2010	6	0	1	1	0	0	8
2011	4	0	1	2	0	0	7
2012	2	0	3	1	0	2	8
2013	4	1	0	0	0	3	8
2014	10	0	1	1	1	5	18
2015	19	1	5	3	1	4	33
2016	41	7	1	7	1	19	76
2017	78	10	25	8	12	52	185
2018	149	53	16	16	11	83	328
2019	226	95	28	27	21	109	506
2020	291	163	29	46	23	121	673
2021	496	310	69	59	56	220	1,210
Total	1,326	640	179	171	126	618	3,060
Mean	110.5a	53.3ab	14.9ab	14.3ab	10.5ab	51.5	255.0
Std dev	155.4	95.6	20.5	19.7	16.7	69.5	374.4

**Figure 1:**
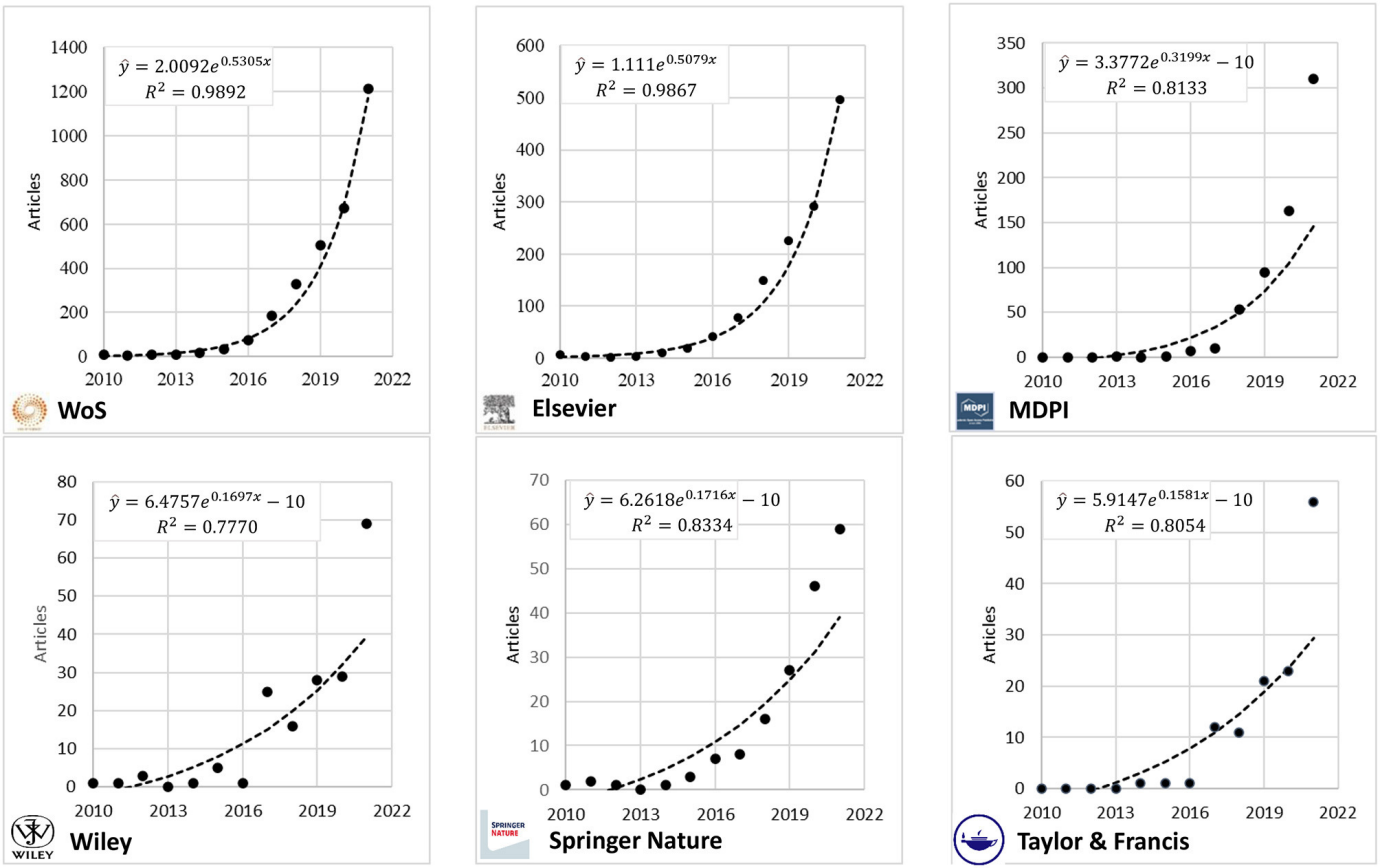
Evolution of scientific production on the circular economy published in the Web of Science according to main publishers.

The scientific production of original articles corresponds to a count variable; moreover, it is evident that the variance (square of the standard deviation) is much higher than the mean, an indicator of the presence of overdispersion, which was confirmed by a comparison performed using negative binomial regression (X2 = 4,110.34, *p* = 0.000), and there is also a difference in the production of Elsevier (*p* = 0.001) and MDPI (*p* = 0.017), but not of Wiley (*p* = 0.607) and Springer Nature (*p* = 0.655), with respect to Taylor & Francis being taken as the reference. The evolution over time of publications on the circular economy is shown in Figure 1, separately for publications in the WoS and the publishers Elsevier and MDPI, and together for the publishers Wiley, Springer Nature, and Taylor & Francis. The trend for publications in the WoS and Elsevier was estimated using the log-log regression model in Excel, as follows:

Web of Science (WoS)


$$\begin{array}{c} \hat{y}\;=\;2.0092e^{0.5305x}\\ R^{2}\;=\;0.9892 \end{array}$$

Elsevier


$$\begin{array}{c} \hat{y}\;=\;1.111e^{0.5079x}\\ R^{2}\;=\;0.9867 \end{array}$$

where y is the annual scientific production and x (2010: x = 1, 2011: x = 2, etc.) corresponds to the year of production. The coefficients of determination R2 of the log-linearized models were 98.92% and 98.67% in the WoS and Elsevier, respectively. The absence of scientific production published in some publishers in some periods made it impossible to estimate their corresponding regression model unless other strategies had been established to do so, but this is not the purpose of the present study. Therefore, we assumed a logarithmic trend for publications in MDPI, Wiley, Springer Nature, and Taylor & Francis, even though publications in Wiley showed many ups and downs.

Assuming a logarithmic growth of the scientific production on the circular economy means that the growth rate is not constant each year, having had higher growth in publications on the subject during the pandemic. In the WoS, there was an increase of only 33% in the period 2019–2020 and 79.8% in the period 2020–2021, very similar to that presented in Elsevier (28.8 and 70.4%, respectively), and contrary to Springer Nature (70.4 and 28.3%, respectively). In contrast, at MDPI there was a constant increase (71.6 and 90.2%), but Wiley (3.6 and 137.9%) and Taylor & Francis (9.5 and 143.5%) changed from a small to large annual growth. Using logarithmic models, it was estimated that by 2022 there would be 1,987 articles in the WoS, of which 819 would be published in Elsevier. The reality showed that, up to October 2022, 1,093 articles had already been published in the WoS, with 387 published in Elsevier, below the growth expectations.

### Characteristics of scientific production

The main characteristics of scientific production in the period 2010–2022 (up to 21 October 2022) according to main publishers are presented in Table 2. First, we have the number of times the articles were cited and then the possible factors associated with the citations. As for the citations of the articles in the WoS, the highest corresponded to Wiley (25.5 ± 62.0 citations) and Elsevier (28.1 ± 54.5 citations), followed by Taylor & Francis (15 ± 33.2 citations), Springer Nature (14.0 ± 53.6 citations). The mean number of citations is influenced by the non-citations of some of these articles, which reached 23.8% in Taylor & Francis, 20.8% in Springer Nature, 19.6% in MDPI, 19% in Wiley, and only 9.4% in Elsevier. The non-citation of articles is an important element to take into account in the choice of the regression model for the analysis of factors associated with citations; likewise, the fact that the variance (square of the standard deviation) is much greater than the mean number of citations constitutes another relevant aspect in the modeling in reference. Conversely, the mean number of citations and also the variance are influenced by the maximum number of citations an article can have. In Elsevier, an article has reached up to 1,048 citations, and 851 in Springer Nature, 441 in Wiley, 333 in MDPI, and 288 in Taylor & Francis.

**Table 2:** Characteristics of the scientific production on the circular economy published in the Web of Science in main publishers, 2010–2022.

**Publisher**	**Variables**	**Min**.	**Max**.	**Mean**	**Std. Deviation**	**Non citation (%)**
Elsevier (*N* = 1,713)	Times cited	0	1,048	28.1	54.5	9.4
	Cited reference count	2	331	69.8	33.7	
	Number of pages	2	58	12.4	3.7	
	Number of authors	1	24	4.0	2.1	
	Number of research areas	1	4	2.3	0.7	
	Number of author keywords	0	16	5.4	1.4	
	Funding orgs	0	1	0.7	0.5	
MDPI (*N* = 878)	Times cited	0	333	9.1	19.5	19.6
	Cited reference count	6	217	64.8	30.7	
	Number of pages	3	68	19.7	6.0	
	Number of authors	1	17	3.9	1.9	
	Number of research areas	1	4	1.9	0.6	
	Number of author keywords	0	13	5.8	1.7	
	Funding orgs	0	1	0.7	0.5	
Wiley (*N* = 290)	Times cited	0	441	28.5	62.0	19.0
	Cited reference count	0	202	73.8	34.0	
	Number of pages	4	36	15.1	5.1	
	Number of authors	1	11	3.7	1.8	
	Number of research areas	1	3	2.4	0.7	
	Number of author keywords	0	10	5.9	1.2	
	Funding orgs	0	1	0.6	0.5	
Springer Nature (*N* = 287)	Times cited	0	851	14.0	53.6	20.2
	Cited reference count	4	210	62.3	33.6	
	Number of pages	3	53	17.0	7.4	
	Number of authors	1	16	3.9	2.4	
	Number of research areas	1	4	1.6	0.8	
	Number of author keywords	0	10	5.2	1.8	
	Funding orgs	0	1	0.6	0.5	
Taylor & Francis (*N* = 185)	Times cited	0	288	15.0	33.2	23.8
	Cited reference count	0	207	67.1	34.0	
	Number of pages	3	41	17.2	6.2	
	Number of authors	1	13	3.0	1.7	
	Number of research areas	1	5	1.9	1.1	
	Number of author keywords	0	10	5.2	1.5	
	Funding orgs	0	1	0.5	0.5	

Table 2 presents the descriptive statistics of the possible factors associated with citations, only mentioning certain unexpected aspects. Articles without any references (Wiley and Taylor & Francis) or many references (Elsevier, 331; MDPI, 217; Springer Nature 210: Taylor & Francis, 207; and Wiley, 202). More than 50 pages (MDPI, 68; Elsevier, 58; and Springer Nature, 53) or more than 20 authors (Elsevier, 24). Articles without author keywords in all publishers. In addition, articles in which funding sources were stated represented 70% of Elsevier and MDPI publications, 60% of Wiley and Springer Nature, and 50% of Taylor & Francis, respectively.

### Factors associated with citations

Negative binomial regression models to evaluate the factors associated with citations and non-citations of circular economy articles from the top five publishers are shown in Table 3. Modeling is necessary to contrast the effect of the factors, being considered positive if they are directly associated with the citations of the articles and negative if they are associated with a lower number of citations. Model 1 was estimated by default in Stata. The selected factors positively influencing citations were the number of references cited in the article (β = 0.00914, *p* = 0.000) and the number of authors (β = 0.03991, *p* = 0.000). On the other hand, articles in which a financial institution is stated presented fewer citations (β = −0.07791, *p* = 0.034). Furthermore, articles published in the journals of the publishers Elsevier (β = 0.37267, *p* = 0.000) and Wiley (β = 0.39543, *p* = 0.000) received more citations than those published in Taylor & Francis, but articles from Taylor & Francis received more citations than those from MDPI (β = −0.39799, *p* = 0.000), and there was no difference from Springer Nature in the citations received (*p* = 0.536).

**Table 3:** Negative binomial regression models for the number of times cited and non-citations of circular economy articles in the Web of Science from major publishers, 2010–2022.

	**Model 1:**			**Model 2: Robust**			**Model 3: Robust**		
	**Coef**.	**Std. Err**.	***p***	**Coef**.	**Std. Err**.	***p***	**Coef**.	**Std. Err**.	***p***
**Times cited**									
Cited reference count	0.00914	0.00055	0.000	0.00914	0.00069	0.000	0.00923	0.00069	0.000
Number of authors	0.03991	0.00838	0.000	0.03991	0.01153	0.001	0.03757	0.01147	0.001
Funding orgs	−0.07791	0.03678	0.034	−0.07791	0.04607	0.091			
Publisher (reference: Taylor & Francis)							
Elsevier	0.37267	0.07930	0.000	0.37267	0.13220	0.005	0.36151	0.13461	0.007
MDPI	−0.39799	0.08400	0.000	−0.39799	0.13586	0.003	−0.41480	0.13891	0.003
Wiley	0.39543	0.09494	0.000	0.39543	0.15745	0.012	0.38537	0.15957	0.016
Springer nature	−0.06036	0.09747	0.536	−0.06036	0.16135	0.708	−0.06488	0.16280	0.690
**Publication year (Reference: 2019)**									
2010	1.43501	0.31932	0.000	1.43501	0.18634	0.000	1.41910	0.18314	0.000
2011	1.05840	0.34125	0.002	1.05840	0.27730	0.000	1.08262	0.28418	0.000
2012	1.63339	0.36743	0.000	1.63339	0.44332	0.000	1.63329	0.43512	0.000
2013	0.51897	0.40585	0.201	0.51897	0.30754	0.092	0.51317	0.30292	0.090
2014	1.21323	0.25244	0.000	1.21323	0.20755	0.000	1.19415	0.20593	0.000
2015	0.70779	0.17357	0.000	0.70779	0.22463	0.002	0.69564	0.22174	0.002
2016	0.83346	0.12749	0.000	0.83346	0.17755	0.000	0.83977	0.17896	0.000
2017	0.80634	0.09114	0.000	0.80634	0.12081	0.000	0.79848	0.11991	0.000
2018	0.55882	0.07348	0.000	0.55882	0.09714	0.000	0.56164	0.09857	0.000
2020	−0.43246	0.06036	0.000	−0.43246	0.06687	0.000	−0.42990	0.06684	0.000
2021	−1.35932	0.05523	0.000	−1.35932	0.06525	0.000	−1.35534	0.06526	0.000
2022	−2.93916	0.06137	0.000	−2.93916	0.08276	0.000	−2.93273	0.08281	0.000
cons	2.59354	0.09981	0.000	2.59354	0.15100	0.000	2.55376	0.14704	0.000

On the other hand, it is expected that the older the article is, the more citations it receives, but this was not always the case. Although articles published in the period 2010–2012 received more citations than those published in 2019 (pre-pandemic COVID-19), the older articles did not necessarily receive more citations. No difference was found in the number of citations with the articles published in 2013 (*p* = 0.201). However, again there were more citations in the articles published in the period 2014–2018 than in 2019. More recent articles from 2020–2022 (published during the pandemic) were cited fewer times than those published in 2019.

It is possible that certain articles published in one period coincide in the references used in another period, in that some authors or institutions funding them are the same and also that they are published in the same journals or in journals of the same publisher; this determined an evaluation of the robustness of the model and the factors, which are shown in models 2 and 3 in Table 1. As for the factors number of references and number of authors, they confirm their positive influence on the number of citations of the articles in robust model 2, and as a negative factor if they state a source of financing, but at 10% (*p* = 0.091). Model 2 confirms the differences in the number of citations received by articles from Elsevier and Wiley publishers indicated above, and also from MDPI with respect to those from Taylor & Francis. Compared to 2019, the model confirms the differences in citations for each year, and some differences with respect to 2013, but at 10% (*p* = 0.092). The differences found in model 2 were maintained in model 3 even though the statement of a financial institution was disregarded in the model.

### Factors associated with non-citations

We found that about 20% of articles in the WoS on the circular economy were not cited, with the exception of Elsevier articles, of which only 9.4% were not cited, which determined the inclusion of zero-inflated negative binomial regression models, considering a logit model for non-citations, but at the same time including analysis of citations, as shown in Table 4.

**Table 4:** Zero-inflated negative binomial regression models for the number of times cited and non-citations of circular economy articles in the Web of Science from major publishers, 2010–2022.

	**Model 4:**			**Model 5: Robust**			**Model 3: Robust**		
	**Coef**.	**Std. Err**.	**p**	**Coef**.	**Std. Err**.	**p**	**Coef**.	**Std. Err**.	**p**
**Times cited**									
Cited reference count	0.00905	0.00056	0.000	0.00905	0.00069	0.000	0.00914	0.00069	0.000
Number of authors	0.04029	0.00837	0.000	0.04029	0.01153	0.000	0.03790	0.01146	0.001
Funding orgs	−0.07930	0.03673	0.031	−0.07930	0.04607	0.085			
**Publisher (reference: Taylor & Francis)**									
Elsevier	0.37194	0.07926	0.000	0.37194	0.13235	0.005	0.36057	0.13478	0.007
MDPI	−0.39964	0.08395	0.000	−0.39964	0.13603	0.003	−0.41673	0.13910	0.003
Wiley	0.39649	0.09488	0.000	0.39649	0.15761	0.012	0.38615	0.15976	0.016
Springer Nature	−0.06266	0.09737	0.520	−0.06266	0.16152	0.698	−0.06726	0.16299	0.680
**Publication year (reference: 2019)**									
2010	1.43330	0.31849	0.000	1.43330	0.18655	0.000	1.41714	0.18329	0.000
2011	1.05710	0.34037	0.002	1.05710	0.27732	0.000	1.08180	0.28433	0.000
2012	1.63090	0.36647	0.000	1.63090	0.44309	0.000	1.63089	0.43475	0.000
2013	0.51609	0.40482	0.202	0.51609	0.30755	0.093	0.51023	0.30286	0.092
2014	1.21262	0.25178	0.000	1.21262	0.20734	0.000	1.19320	0.20570	0.000
2015	0.70682	0.17313	0.000	0.70682	0.22473	0.002	0.69445	0.22178	0.002
2016	0.83294	0.12716	0.000	0.83294	0.17747	0.000	0.83933	0.17889	0.000
2017	0.80634	0.09091	0.000	0.80634	0.12107	0.000	0.79834	0.12014	0.000
2018	0.56246	0.07339	0.000	0.56246	0.09710	0.000	0.56522	0.09856	0.000
2020	−0.43079	0.06024	0.000	−0.43079	0.06686	0.000	−0.42821	0.06682	0.000
2021	−1.35659	0.05514	0.000	−1.35659	0.06525	0.000	−1.35258	0.06525	0.000
2022	−2.93690	0.06127	0.000	−2.93690	0.08277	0.000	−2.93041	0.08283	0.000
cons	2.59933	0.09976	0.000	2.5993	0.15124	0.000	2.55880	0.14729	0.000
**inflate**									
Cited reference count	−0.19731	0.07310	0.007	−0.19731	0.03490	0.000	−0.19795	0.03492	0.000
Cons	−0.88551	1.03692	0.393	−0.8855053	1.01086	0.381	−0.8976701	1.02204	0.380

In relation to non-citations (inflate), in model 4, only the number of references cited in the article was recognized as a negative factor to non-citations (β = −0.19731, *p* = 0.007), i.e., it favors the citation of articles. The effect of the number of references used was confirmed by robust models 5 and 6, the latter omitting the analysis of citations regarding the statement of financial institutions. As for article citations, the zero-inflated models 4–6 confirmed the effect of the factors found in models 1–3, except for the statement of financial institutions, which only reaches significance at 10% (*p* = 0.091). Similarly, they confirmed differences between publishers and citations according to age, except that, at 10%, differences could already be found in citations from 2013 with 2019 both in model 5 (*p* = 0.092) and model 6 (*p* = 0.090). Models 5 and 6 gave robustness to the selected factors. It is logical that the possibility of citations of an article increases with the passage of time, which is why the coefficients had a positive sign between 2010–2018 and negative in the period 2020–2022. However, no difference was found between the citations of those published in 2013 and 2019, which is explained by the fact that those published in 2013 have on average the lowest number of citations in that period (47.9 ± 46.4 citations) and not by other factors such as the number of articles published each year.

### Model performance

Table 5 shows the results of tests to compare the negative binomial regression (models 1–3) and zero-inflated negative binomial regression (models 4–6) models. The deviancy analysis reveals that together the selected factors explain the number of citations of the articles of the five publishers under study, using the X2 test of the likelihood ratio in the default models or the Wald test in the robust models. The pseudo R2 = 0.131 in the negative binomial regression models is not an indicator of zero-inflated models. The models revealed the existence of overdispersion in the number of citations (*p* = 0.000, in both cases); this determined that a Poisson regression model would not be adequate to analyze the citations and non-citations in the articles of the publishers on the subject of study found in the WoS. This confirms that negative binomial regression models are an alternative to Poisson regression models with the same configuration. Both the citation and non-citation of articles are indicators of the quality of publications, being of special interest that authors, journals, and publishers are interested in increasing the citations of articles and avoiding non-citations. This is relevant for considering zero-inflated regression models. In this case, even though the Vuong test did not confirm the need (*p* = 0.117 > 0.05), it allowed the zero-inflated models to rescue the number of references cited in the articles as a factor that protects against the non-citation of articles on the circular economy, whose robust models present similar information criteria AIC (22581 and 22585) and BIC (22710 and 22719). Furthermore, the models validated the selected factors. The Vuong test allowed for a structural comparison in the modeling and whether or not to consider the excessive number of zeros.

**Table 5:** Benefits of negative binomial and zero-inflated regression models.

	**Negative binomial regression**			**Zero-inflated negative binomial regression**		
	**Model 1**	**Model 2**	**Model 3**	**Model 4**	**Model 5**	**Model 6**
**Deviance goodness-of-fit test**						
LR (or Wald): X^2^	3401	3238	3224	3404	3229	3215
p	0.000	0.000	0.000	0.000	0.000	0.000
Pseudo R^2^	0.131	0.131	0.131			
Overdispersion: Alpha	0.788	0.788	0.789	0.784	0.784	0.785
X^2^	5.00E+04			5.00E+04		
p	0.000			0.000		
**Zero-inflated: Vuong test**						
z				0.930		
p				0.177		
**Information criterion**						
AIC	22581	22581	22584	22585	22582	22585
BIC	22710	22710	22706	22719	22723	22719

In the negative binomial regression, the overdispersion is tested by the Ho hypothesis: μ 6 = 0, which is proof of a variability greater than that assumed by the Poisson regression (variability equal to the mean μ).

## Discussion

It is relevant that Elsevier is the leader of publications on the circular economy, with twice as many articles as the publisher in second place, MDPI. The difference between the 3 publishers is very marked. Research into the circular economy will continue to grow and knowing the publishers that publish the most allows authors to choose those journals that most accept articles related to the circular economy; it is also relevant to recognize that there are other journals that have had volumes in which they did not publish articles on the circular economy, which also suggests that publishers can reflect on strategies to encourage more publications in this field, such as the generation of special issues focused on circularity.

Given that the increase in the publication of articles on the circular economy in 2019–2020 was 33% and 79.8% in 2020–2021, it can be understood that there was growth in all journals. However, surprisingly, this was not the case. In MDPI, the growth was 25%; in Elsevier, the growth almost tripled; but in Springer Nature, it was reduced to one third. What was very remarkable was the growth of Wiley of 35 times and Taylor and Francis of 14 times. The non-citation of articles can indicate several factors, such as a lack of relevance to the current research topic or an insufficient contribution to the existing literature. The inclusion of such articles in the analysis can negatively impact the accuracy and validity of regression analysis results. Therefore, it is crucial to exclude these articles or account for their potential impact when selecting a regression model. The regression model chosen for the analysis should be able to accurately predict the citation behavior of relevant articles while eliminating the influence of non-cited articles. This is to ensure that the results of the regression analysis are reliable and contribute to science. For data analysis, researchers can use various regression techniques to analyze the citation behavior of articles considering the articles not cited. These techniques have different bene?ts for evaluating these elements whose data appear dynamically.

There was a skewed distribution of the citation count where it is possible to identify the articles that are little cited and, likewise, those with many citations which generate a relevant impact on the variance of the distribution of citations. Both extremes have an impact on the calculation. It should always be kept in mind that outliers can influence statistical analyses such as regressions. Another aspect to be considered in the calculation is that there are some very specific articles with a very high citation amount, which will also generate an alteration of the results. To solve this problem, optional methods such as non-parametric regression can be used, which are much less influenced by these extreme values. Researchers should be aware of these data limitations in order to make a more accurate interpretation.

Citation counts of an author's publications are a traditional metric that shows how much impact these articles are having. Thus, it is considered more reputable to have more citations and that these citations are in high impact journals. In this study, we have used negative binomial regression models in order to identify the factors that have a significant impact on the number of citations of circular economy articles from the top five publishers. The study highlights that the number of references cited in the article positively influences the number of citations an article receives. This result is consistent with previous research, which guides support for research in universities. Google Scholar's algorithm weights publications according to their citations, ranking them first in searches, which will lead to them being read as a priority, increasing their likelihood of future citations.

A relevant result is that the number of citations is an indicator of the quality of the content of the article; likewise, articles that cite more articles may be perceived as more rigorous and credible by the scientific community since there would have been a greater search to substantiate the conceptual aspects and compare the results obtained. The findings of the study have several implications for researchers. Authors of articles should conduct detailed literature reviews so that the most relevant and pertinent articles can be cited. The quality of the references cited in an article may also have an influence on its impact on the scientific community and future citations. Editors can use the results to generate guidelines to guide researchers when writing high quality articles that can make their journals more impactful. It is also a good opportunity to highlight the value of literature reviews in finding knowledge gaps and generating innovative research to solve specific problems in a country or region.

Policy makers can use these results to design research policies and financial support for research in the field of the circular economy, coordinating with universities, companies, and state institutions. Some limitations of the study include that the study only analyzed articles on the circular economy from the top five publishers.

The analysis of the factors that influence citations of scientific articles reveals patterns and trends in the way research in the field of the circular economy is received and valued, which contributes to the orientation of future research. These factors could include the content of the article, the quality of the research, the methodology used, the novelty of the ideas presented, and the clarity of the communication, among others. Identifying these factors provides useful information for researchers wishing to increase the impact of their work and for journal editors seeking to publish high quality research. It is needed to recognize that the study can be used as a foundation for future research for comparison in a few years to see the variation of citations and trends of circular economy research. Understanding which characteristics and approaches of circular economy scientific articles tend to receive the most citations can help researchers improve the quality and relevance of their work. This can drive the production of more robust and meaningful research in the field, which in turn will contribute to the advancement of knowledge and the implementation of sustainable practices. This type of study not only sheds light on the specific topic of the circular economy but can also provide knowledge and methodologies that are transferable to other research fields. Understanding the factors that influence the citation of scientific articles is a common concern across disciplines, so the methods and results of this study could inspire similar research in other areas.

Therefore, the results may not be generalizable to other research fields or publishers; furthermore, the study is limited to quantitative analysis and does not consider qualitative factors that may influence the number of citations an article receives.

## Conclusion

The study highlights the importance of citing relevant studies and conducting thorough literature reviews to increase the likelihood of an article being cited. The number of references cited in an article may also reflect the quality of research and its credibility. These findings have several implications for researchers, publishers, and policymakers, and can be used to enhance the impact of research in the circular economy and other fields of study. However, the study has some limitations such as a lack of specification of the gap in the literature and lack of a literature review section, and future research should account for qualitative factors that may influence the impact of an article beyond the number of citations it receives.

## Data availability statement

The original contributions presented in the study are included in the article/supplementary material, further inquiries can be directed to the corresponding author.

## Author contributions

CM-M and DT-P: Conceptualization, Methodology, Software, Validation, Formal analysis, Investigation, Resources, Data curation. CM-M, DT-P, AA-R, SD-A-A, and JY: Writing—original draft preparation, Writing—review and editing, Visualization. All authors contributed to the article and approved the submitted version.
